# The New Red Cross Society

**Published:** 1905-07-29

**Authors:** 


### The Hospital.

A Journal of

The Medical Sciences and Hospital Administration.

Vol. XXXVIII.—No. 983. SATURDAY, JULY 29, 1905.

### THE NEW RED CROSS SOCIETY.

It is known that Her Majesty Queen Alexandra has for many years past desired to secure a central and efficient Red Cross Society, which should be so representative as to guarantee the maximum of assistance being rendered to the Navy and Military Departments in case of war. The first step in the realisation of Her Majesty's ideal was taken at the meeting at Buckingham Palace last week, when the new British Red Cross Society was established. It will be based upon membership, and the members and associates of the Society will be recruited from all classes throughout the Empire. The society will be entirely voluntary, but we presume that in time of war, though not in times of peace, it will be under naval and military control. Providing care is taken to unite all those who are interested in this great work at the outset, and to secure an adequate recognition of each nationality in the United Kingdom, there is every reason to hope for the best results from the new organisation.

The object of the Red Cross Society is to furnish volunteer aid to the sick and wounded of armies in time of war, in accordance with the spirit and conditions of the Geneva Convention. It is essential that in all matters of voluntary relief the Society shall be in accord with the military and naval authorities, and shall constitute a link and basis of communication between the people of the United Kingdom and the army and navy. It will also have to keep in close touch with all similar societies established in any of the 44 nations which have now joined the Geneva Convention. In times of peace the new Red Cross Society ought to be made the means of applying relief in mitigating the sufferings caused by pestilence, famine, fire, floods, and other great national calamities. The necessity of being prepared for emergencies has been too often demonstrated to require argument. The first duty of the executive committee of the new society therefore must be to secure that its machinery will be ready to be put in operation, immediately, in time of war or calamity, and that its personnel shall be so efficient and spirited as to win universal confidence.

It is essential to success that due regard shall be paid to the susceptibilities of each nationality. The work is at present being carried out in Ireland by the Society of St. Patrick, and in Scotland by the Society of St. Andrew, the latter of which is so admirably equipped and administered at the present time as to be one of the best, if not the very best, of Red Cross Societies. We understand that some friction may have been caused already for the want of a little tact in regard to one or two matters where more frankness and consideration might have been shown with advantage. It should be perfectly clear to any one possessed of the ability to justify their taking a prominent part in the new Red Cross Society, that any attempt to wipe out the Society of St. Andrew or the Society of St. Patrick, and to absorb their funds, must fail, and deserves to fail. If ever the ideal of Queen Alexandra is to be attained in this country national susceptibilities must be safeguarded, for otherwise the organisations in Scotland and Ireland for example must lack vigour and fail to secure the support of the people.

The first duty of the new Red Cross Society is to organise. Organisation is a matter of experience and knowledge, and cannot be attained by leaving it to the mercy of a few amateurs, or allowing it to drift into the hands of two or three ladies who, however zealous, may not possess the necessary knowledge. Each county should have its branch society, so that every person who desires to become a member may readily do so. In this way, and in this way alone, is it possible to awaken throughout the United Kingdom and Ireland a keen, individual interest in the objects and work of the Red Cross Society. Care should be taken to provide that volunteers should everywhere be recruited; that they should be trained for the work by competent inspectors, and that once a year there should be a general assembly of all the members in each county, with the object of testing the average attainment and efficiency both of the organisation and its personnel. The central executive must start initially the organisation in the counties, and the county executives be entrusted with the duty of enrolling practitioners and nurses for Red Cross service in time of war or national disaster. The latter should report annually through the Central Council to the Army and Navy Medical Department the numbers enrolled by each branch. Every branch should have an efficient secretary, either paid or unpaid, and it is desirable that arrangements should be made with the War Office and Admiralty as to the salaries which will be paid by these departments to both practitioners and nurses per annum, in case their services should be utilised for Red Cross work at any time. It is also essential that each county should be so organised as to place within the knowledge of the executive and its secretary accurate details as to where hospital relief supplies can be obtained at the shortest notice. A hospital unit should be established in each county after conference with the Army Medical Department, and experiments should be made in preparing the personnel and supplies needed for a field hospital of fifty beds, so that each county or sub-division of a county may, where necessary, at short notice, be able to provide such a hospital fully equipped for active service. It will be the duty of the executive to determine the annual subscription which shall constitute membership and the capital sum to be paid for life membership, and to decide how such money shall be apportioned between the local and central organisations of the society. These are some of the main principles of organisation which should receive early attention.

